# Total Synthesis of Lignan Lactone (–)-Hinokinin

**DOI:** 10.1007/s13659-015-0073-3

**Published:** 2015-10-12

**Authors:** Qi-Long Zhou, Hui-Jing Wang, Pei Tang, Hao Song, Yong Qin

**Affiliations:** Innovative Drug Research Centre, Chongqing University, Chongqing, 401331 China; Key Laboratory of Drug Targeting and Drug Delivery Systems of the Ministry of Education, West China School of Pharmacy, Sichuan University, Chengdu, 610041 China

**Keywords:** Lignan, Hinokinin, Total synthesis, Cascade reaction

## Abstract

This research paper is aimed at studying the total synthesis of pharmacologically active lignan (–)-hinokinin. The synthesis features a three-step cascade reaction involving highly stereoselective Michael addition, anion-oxidative hydroxylation, and oxygen anion cyclization to construct the pivotal butyrolactonimidate intermediate.

**Electronic supplementary material:**

The online version of this article (doi:10.1007/s13659-015-0073-3) contains supplementary material, which is available to authorized users.

## Introduction

**Fig. 1:**
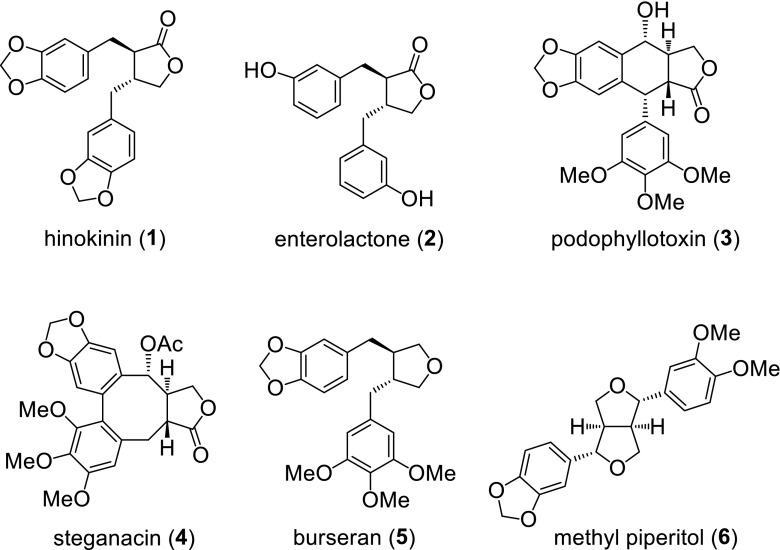
Representive lignans with pharmacological activities

Lignans are a large class of natural products that were isolated from many plants [1, 2] (Fig. 1). They display diverse biological activities, especially antiviral and antitumor properties. For example, hinokinin (**1**) [3–5] has been found to exhibit antileukemic, antiviral, antifungal, and pesticidal activities [6–26]. Mammalian lignin enterolactone (**2**), which is formed by the action of intestinal bacteria from plant lignan precursors present in the diet, inhibit breast cancer and colon cancer, and may also inhibit cardiovascular disease [27, 28]. Furthermore, podophyllotoxin (**3**), steganacin (**4**) and tetrahydrofuran lignan burseran (**5**) show strong cytotoxic activity against various cancer cell lines by preventing the normal function of the mitotic spindle [29–36]. In addition, the furofuran lignan methyl piperitol (**6**) possesses platelet activating factor (PAF) antagonist activity [37]. Due to their interesting biological activities, several members of this family of natural products and their analogs have therefore been the target of extensive synthetic research [38–47].

**Scheme 1:**
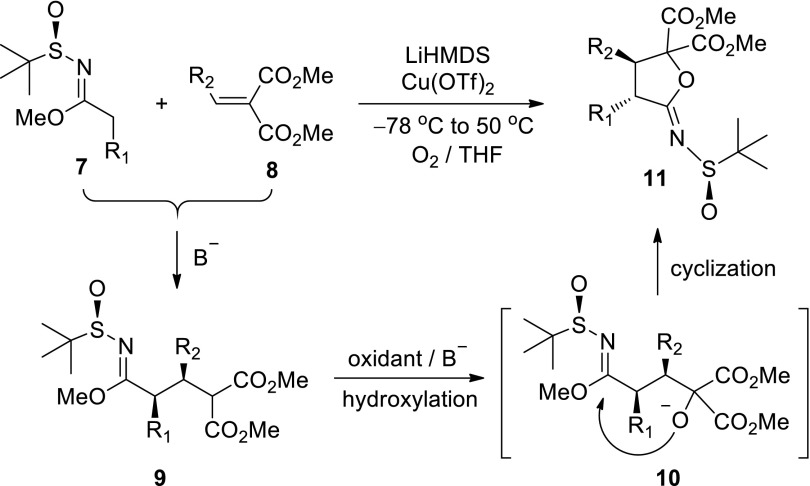
Synthesis of butyrolactonimidates **11** via the three-step cascade reaction

**Scheme 2:**
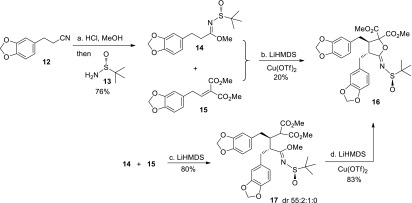
Synthesis of butyrolactonimidate **16**. *Reagents and conditions:* **a** HCl (gas), MeOH, 0 °C, 24 h; MeOH, r.t., 48 h; then (*R*)-*tert*-butanesulfinamide **13**, *p*-TsOH (0.05 equiv), neat, 60 °C, 24 h, 76 % in 2 steps; **b** LiHMDS (5.0 equiv), THF, −78 °C, 0.5 h, then Cu(OTf)_2_ (5.0 equiv), −78 to 60 °C, 24 h, 20 %. **c** LiHMDS (2.2 equiv), THF, −78 °C, 12 h, 80 %; **d** LiHMDS (3.3 equiv), THF, −78 °C, 0.5 h, then Cu(OTf)_2_ (5.0 equiv), −78 to 60 °C, 24 h, 83 %. *LiHMDS* Lithium hexamethyldisilazide, *THF* tetrahydrofuran

**Scheme 3:**
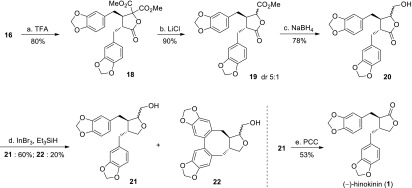
Synthesis of (–)-hinokinin (**1**). *Reagents and conditions:* **a** TFA (10.0 equiv), CH_2_Cl_2_, 0 °C to r.t., 80 %; **b** LiCl (5.0 equiv), DMSO, 100 °C, 8 h, 90 %; **c** NaBH_4_ (2.5 equiv), MeOH, 0 °C, 10 h, 78 %; **d** InBr_3_ (0.05 equiv), Et_3_SiH (5 equiv), CHCl_3_, sealed tube, 60 °C, 2 h, 60 % for **21**, 20 % for **22**; **e** PCC (5.0 equiv), PhMe, 80 °C, 5 h, 53 %. *LiHMDS* Lithium hexamethyldisilazide, *THF* tetrahydrofuran, *TFA* trifluoroacetic acid, *DMSO* dimethyl sulfoxide, *PCC* pyridinium chlorochromate

We recently developed a one-pot, three-step cascade reaction from enantiomerically pure (*R*)-*N*-*tert*-butanesulfinyl imidates **7** and *α*,*β*-unsaturated diesters **8** [48] to generate butyrolactonimidates **11** (Scheme 1). This reaction proceeded through highly stereoselective Michael addition (**7**–**9**), followed by anion-oxidative hydroxylation (**9**–**10**) and oxygen anion cyclization (**10**–**11**). The synthesized butyrolactonimidates **11** are versatile intermediates for preparing substituted butyrolactones and furans. We also used this approach to achieve the concise total synthesis of natural product (–)-nephrosteranic acid [48]. In this paper, we report the total synthesis of lignan lactone (–)-hinokinin **1** as shown in Schemes 2 and 3.

## Results and Discussion

We began our synthesis with the preparation of enantiopure (*R*)-*N*-*tert*-butanesulfinyl imidate **14** (Scheme 2): treatment of the known nitrile **12** [49, 50] with gaseous HCl in methanol yielded a good amount of trimethylorthoester intermediate [51], subsequent condensation of (*R*)-*tert*-butanesulfinamide **13** and the corresponding trimethylorthoester with a catalytic amount of *p*-TsOH without solvent afforded chiral (*R*)-*N*-*tert*-butanesulfinyl imidate **14** in 76 % overall yield [52–54]. With the *N*-sulfinyl imidate **14** in hand, we focused our attention on the construction of the pivotal butyrolactonimidate, as shown in Scheme 2. Firstly, we performed the one pot protocol between **14** and the known *α,β*-unsaturated diester **15** [55, 56] under optimal condition [48] {LiHMDS (5.0 equiv), −78 °C; Cu(OTf)_2_ (5.0 equiv), −78 to 60 °C} to afford the desired butyrolactonimidate **16** in low yield (20 %), due to the isomerization of double bond in **15** under excess LiHMDS. To our delight, the stepwise procedure provided a satisfactory result. Indeed, a LiHMDS-mediated highly stereoselective Michael addition of **14**–**15** produced adduct **17** in 80 % yield as the dominant stereoisomer (dr 55:2:1:0 by LC–MS). Next, the resulting Michael adduct underwent the Cu(OTf)_2_-mediated anion-oxidative hydroxylation and oxygen anion cyclization to deliver **16** in 83 % yield.

With the pivotal butyrolactonimidate **16** in hand, the synthesis of natural product (–)-hinokinin **1** was investigated (Scheme 3). The chiral *tert*-butylsulfinyl moiety in **16** was readily removed by TFA in CH_2_Cl_2_ to afford butyrolactone **18** in 80 % yield. Krapcho demethoxycarbonylation of **18** with LiCl in DMSO afforded **19** in 90 % yield as a 5:1 mixture of epimers [57–59], as determined by ^1^H NMR spectroscopy. The mixture of epimers could not be separated by chromatography, however this would prove to be inconsequential since this carbon would become sp2 hybridized in subsequent steps. Reduction of the ester group in mixture **19** with NaBH_4_ in MeOH gave alcohol **20** in 78 % yield. Subsequent reduction of the lactone group in **20** using InBr_3_ and Et_3_SiH in CHCl_3_ generated the desired furan **21** in 60 % yield as a mixture of epimers [60, 61]. Interestingly, besides furan **21**, further aromatic oxidative coupling proceeded under this condition to deliver compound **22** in 20 % yield, which possessed the tetracyclic scaffold of dibenzocyclooctadiene lignans such as steganacin **4** (Fig. 1). The generation of **22** presumably resulted from the introduction of oxygen under this reductive condition, and the amount of **22** was considerably increased (TLC monitoring) when oxygen was intentionally bubbled into the reaction tube. Finally, heating of **21** with excess PCC in toluene completed the total synthesis of lignan lactone (–)-hinokinin **1** in acceptable yield [62, 63], which displayed identical spectral properties to the reported data [3–5, 18, 21, 24, 26].

## Conclusion

In summary, we accomplished the total synthesis of the lignan lactone (–)-hinokinin **1** in 8 steps. The synthesis is based on a three-step cascade reaction involving highly stereoselective Michael addition, anion-oxidative hydroxylation, and oxygen anion cyclization to construct the pivotal butyrolactonimidate **16**. The strategy we developed may find use in the synthesis of other pharmacologically active lignans.

## Experiment Section

All commercially available reagents were used without further purification. All solvents were dried and distilled before use; THF was distilled from sodium. Chromatography was conducted by using 200–300 mesh silica gel. Petroleum ether refers to the 60–90 °C boiling fraction. All new compounds gave satisfactory spectroscopic analyses (IR, ^1^H NMR, ^13^C NMR, HRMS). IR spectra were recorded on a FT IR spectrometer. NMR spectra were recorded on 600/400 MHz NMR spectrometers. HRMS spectra were obtained by the ESI-TOF method. Experimental conditions and spectral data were published previously for compounds **12** [49, 50] and **15** [55, 56].

### Methyl (*R*,*Z*)-3-(benzo[*d*][1,3]dioxol-5-yl)-*N*-(*tert*-butylsulfinyl)propanimidate (**14**)

1. A mixture of the chosen nitrile **12** (28.60 mmol, 1.0 equiv) and methanol (37.00 mmol, 1.3 equiv) was charged in a 50 mL flask, and cooled in an ice bath. Gaseous HCl was slowly bubbled into the methanolic solution of the nitrile for 20 min. The resulting mixture was kept at 0 °C for 24 h. Then, the excess of methanol and HCl was removed by washing with Et_2_O, white solid imidate hydrochloride was separated. The product was dried under vacuum at rt, and used as such for the subsequent methanolysis step.
2. At rt, a mixture of methanol (10 mL) and the solid imidate hydrochloride was set to react under stirring for 48 h. A clear solution was obtained. White solid (ammonium chloride) was formed during the reaction. Then, the mixture was filtered to cleavage NH_4_Cl and washed with Et_2_O. The solvent was removed in vacuo to get the colorless oil trimethyl-intermediate (6.5 g, 90 %).
3. Under N_2_, to neat trimethyl-intermediate (9.84 mmol, 2 equiv) was added (*R*)-*tert*-butanesulfinamide **13** (4.92 mmol, 1 equiv) and *p*-TsOH (0.25 mmol, 0.05 equiv). The reaction mixture was kept at 60 °C for 24 h. The resulting residue was purified by column chromatography (silica gel) to give **14** (1.3 g, 84 %) as a colorless oil. $\left[ \alpha \right]_{D}^{23}$ −97.0 (*c* 0.22, CH_2_Cl_2_); ^1^H NMR (400 MHz, CDCl_3_) *δ* 6.64–6.72 (m, 3H), 5.09 (s, 2H), 3.51 (s, 3H), 2.86–2.93 (m, 4H), 1.18 (s, 9H); ^13^C NMR (100 MHz, CDCl_3_) *δ* 175.6, 147.6, 146.0, 133.8, 121.2, 108.8, 108.2, 100.8, 55.8, 54.1, 34.8, 32.1, 21.8; HRMS [M + Na]^+^ calcd for C_15_H_21_NNaO_4_S 334.1083, found 334.1084; IR (KBr) 2948, 1608, 1491, 1443, 1245, 1076, 1040, 926, 810, 750, 591.

### Dimethyl 2-((2*R*,3*R*,*Z*)-1-(benzo[*d*][1,3]dioxol-5-yl)-3-(benzo[*d*][1,3]dioxol-5-ylmethyl)-4-(((*R*)-*tert*-butylsulfinyl)imino)-4-methoxybutan-2-yl)malonate (**17**)

Under N_2_, to a solution of **14** (1.90 mmol, 0.95 equiv) in dry THF (100 mL) was added LiHMDS (1 M in THF, 4.20 mmol, 2.2 equiv) at −78 °C. After the resulting solution was maintained at −78 °C for 30 min, a solution of **15** (2.00 mmol, 1.0 equiv) in THF (1 mL) was slowly added for 10 h. The resulting solution was maintained at −78 °C for another 12 h. The dr values for the first Michael addition anaylsed by LC–Ms. After the reaction was completed, the solution was quenched at −78 °C by pouring into aqueous NH_4_Cl (50 mL). The aqueous layer was partitioned with EtOAc (3 × 50 mL). The organic layer was separated, dried (Na_2_SO_4_), filtered, and the solvent was removed in vacuo. Flash chromatography (silica gel) of the crude reaction mixture afforded pure coupled product **17** (900 mg, 80 %). Conditions for LC–MS analysis of Michael addition product: Waters ACOUITY UPLC BEH C_18_, BEH C_18_ (2.1 × 100 mm, 1.7 micron particle size), mobile phase H_2_O/CH_3_CN (80:20); Flow = 0.2 mL/min; Detected by UV at 210 nm; Retention time for stereoisomers: 7.78 min (major), 8.25 min, 8.97 min; Dr 55:1:2:0. **17**: $\left[ \alpha \right]_{D}^{23}$ −44.2 (*c* 0.21, CH_2_Cl_2_); ^1^H NMR (400 MHz, CDCl_3_) *δ* 6.62–6.76 (m, 4H), 6.47–6.49 (m, 2H), 5.91 (s, 2H), 5.86 (s, 2H), 3.81–3.84 (m, 1H), 3.73 (s, 3H), 3.68(s, 3H), 3.67 (s, 3H), 3.59 (d, *J* = 4.0 Hz, 1H), 2.79–3.01 (m, 1H), 2.66–2.69 (m, 4H), 0.96 (s, 9H); ^13^C NMR (100 MHz, CDCl_3_) *δ* 175.2, 169.4, 168.6, 147.5, 146.2, 145.8, 134.0, 132.2, 122.2, 122.0, 109.5, 109.3, 108.2, 108.0, 100.8, 55.6, 53.8, 53.5, 52.5, 52.4, 47.8, 42.6, 36.7, 35.4, 21.6; HRMS [M + Na]^+^ calcd for C_29_H_35_NNaO_10_S 612.1874, found 612.1876; IR (KBr) 2960, 1736, 1606, 1491, 1442, 1248, 1039, 806, 702, 591.

### (3*R*,4*R*,*Z*)-dimethyl 3,4-bis(benzo[*d*][1,3]dioxol-5-ylmethyl)-5-(((*R*)-*tert*-butylsulfinyl)imino)dihydrofuran-2,2(3*H*)-dicarboxylate (**16**)

Under N_2_, to a solution of **17** (1.82 mmol, 1 equiv) in dry THF (200 mL) was added LiHMDS (1 M in THF, 6.00 mmol, 3.3 equiv) at −78 °C. After the resulting solution was maintained at −78 °C for 30 min, the Cu(OTf)_2_ (9.10 mmol, 5.0 equiv) was added at once (exposed to air for seconds). Then the reaction mixture was warmed to ambient temperature slowly and kept at 60 °C and charged with nitrogen balloon for 24 h. After the reaction was completed, the solution was kept in room temperature and quenched by pouring it into aqueous NH_4_Cl (100 mL). The aqueous layer was partitioned with EtOAc (3 × 100 mL). The organic layer was separated and washed with with HCl (1 N, 50 mL), water (50 mL) and aqueous NaHCO_3_ (50 mL), dried (Na_2_SO_4_), filtered, and the solvent was removed in vacuo. Flash chromatography (silica gel) of the crude reaction mixture afforded pure coupled product **16** (860 mg, 83 %). $\left[ \alpha \right]_{D}^{23}$ −77.0 (*c* 0.15, CH_2_Cl_2_); ^1^H NMR (400 MHz, CDCl_3_) *δ* 6.41–6.62 (m, 6H), 5.88–5.94 (m, 4H), 3.89 (s, 3H), 3.84 (s, 3H), 2.61–2.91 (m, 5H), 2.13–2.17 (m, 1H), 1.10 (s, 9H); ^13^C NMR (150 MHz, CDCl_3_) *δ* 171.1, 166.1, 147.4, 146.1, 130.7, 122.4, 122.1, 109.6, 109.1, 108.2, 107.8, 101.1, 100.8, 60.4, 53.9, 53.4, 53.3, 45.8, 30.9, 22.2, 21.03, 14.2; HRMS [M + Na]^+^ calcd for C_28_H_31_NNaO_10_S 596.1561, found 596.1569; IR (KBr) 2962, 2926, 1749, 1665, 1504, 1492, 1445, 1364, 1260, 1088, 1039, 803.

### (3*R*,4*R*)-dimethyl 3,4-bis(benzo[*d*][1,3]dioxol-5-ylmethyl)-5-oxodihydrofuran-2,2(3*H*)-dicarboxylate (**18**)

To a solution of **16** (0.80 mmol, 1.0 equiv) in DCM (30 mL) cooled in an ice-water bath was added TFA (8.00 mmol, 10.0 equiv), and the mixture was stirred at room temperature for 24 h. The reaction was quenched by the addition of sat. NaHCO_3_. The mixture was extracted with EtOAc (30 mL × 3), dried over Na_2_SO_4_ and then concentrated. The resulting residue was purified by column chromatography (silica gel) to give **18** (240 mg, 80 %) as a white solide. $\left[ \alpha \right]_{D}^{23}$ +8.0 (*c* 0.12, CH_2_Cl_2_); ^1^H NMR (400 MHz, CDCl_3_) *δ* 6.72 (d, *J* = 8.4 Hz, 1H), 6.59–6.67 (m, 3H), 6.20–6.22 (m, 2H), 5.97(d, *J* = 4.8 Hz, 2H), 5.91 (d, *J* = 4.6 Hz, 2H), 3.86 (s, 3H), 3.84 (s, 3H), 3.03–3.10 (m, 2H), 2.73-2.85 (m, 2H), 2.44–2.48 (m, 1H), 2.23 (m, 1H); ^13^C NMR (150 MHz, CDCl_3_) *δ* 175.6, 166.8, 166.7, 148.0, 147.5, 146.6, 146.3, 130.9, 130.0, 122.6, 122.1, 109.8, 109.4, 108.3, 108.0, 101.1, 100.9, 85.7, 53.6, 53.4, 46.5, 45.0, 36.6, 34.5; HRMS [M + Na]^+^ calcd for C_24_H_22_NaO_10_ 493.1105, found 493.1107; IR (KBr) 2956, 2924, 1796, 1748, 1492, 1445, 1250, 1175, 1081, 1039, 930, 862, 810, 737, 651.

### (3*R*,4*R*)-methyl 3,4-bis(benzo[*d*][1,3]dioxol-5-ylmethyl)-5-oxotetrahydrofuran-2-carboxylate (**19**)

To a solution of **18** (0.45 mmol, 1.0 equiv) in DMSO (9 mL) was added LiCl (2.25 mmol, 5.0 equiv), and the mixture was stirred at 100 °C for 8 h. The reaction was quenched by the addition of water (10 mL). The mixture was extracted with EtOAc (10 mL × 3), dried over Na_2_SO_4_ and then concentrated. The resulting residue was purified by column chromatography (silica gel) to give yellow oil **19** (167 mg, 90 %) as a mixture (dr 5:1). ^1^H NMR (400 MHz, CDCl_3_) *δ* 6.66 (d, *J* = 7.8 Hz, 2.4H), 6.34–6.52 (m, 4.8H), 5.92 (d, *J* = 6.5 Hz, 4.8H), 4.75 (d, *J* = 7.9 Hz, 0.2H) (minor), 4.53 (d, *J* = 4.6 Hz, 1H) (major), 3.78 (s, 0.6H), 3.74 (d, *J* = 1.8 Hz, 3H), 2.92–2.97 (m, 1.2H), 2.63–2.78 (m, 2.4H), 2.58 (m, 3.6H). ^13^C NMR (100 MHz, CDCl_3_) δ 177.2, 176.7, 170.0, 169.1, 147.8, 146.5, 131.0, 130.8, 130.4, 130.0, 122.3, 122.1, 121.7, 109.6, 109.4, 109.3, 108.9, 108.3, 108.2, 101.1, 78.5, 77.4, 52.7, 52.4, 45.7, 44.9, 44.5, 43.1, 38.4, 35.7, 34.9, 34.4. HRMS [M + Na]^+^ calcd for C_22_H_20_NaO_8_ 435.1050, found 435.1040.

### (3*R*,4*R*)-3,4-bis(benzo[*d*][1,3]dioxol-5-ylmethyl)-5-(hydroxymethyl)dihydrofuran-2(3*H*)-one (**20**)

To a solution of **19** (0.47 mmol, 1.0 eq) in MeOH (17 mL) cooled in an ice-water bath was added NaBH_4_ (2.50 mmol, 2.5 eq), and the mixture was stirred in an ice-water bath for 10 h. The reaction was quenched by the addition of sat. NH_4_Cl (5 mL). The mixture was extracted with EtOAc (10 mL × 5), dried over Na_2_SO_4_ and then concentrated. The resulting residue was purified by column chromatography (silica gel) to give **20** (140 mg, 78 %) as a yellow oil. ^1^H NMR (400 MHz, CDCl_3_) *δ* 6.70 (m, 2.4H), 6.55–6.66 (m, 2.4H), 6.46 (d, *J* = 8.2 Hz, 2.4H), 5.84–5.98 (s, 4.8H), 4.25–4.30 (m, 0.2H), 4.17–4.23 (m, 1H), 3.52 (d, *J* = 12.6 Hz, 1.2H), 3.13 (dd, *J* = 12.9, 4.8 Hz, 1.2H), 2.64–2.84 (m, 2.4H), 2.36–2.50 (m, 4.8H); ^13^C NMR (100 MHz, CDCl_3_) *δ* 177.4, 147.8, 146.4, 132.1, 131.3, 122.3, 121.7, 121.4, 109.5, 108.7, 108.4, 108.3, 108.3, 108.1, 101.0, 83.7, 80.4, 63.1, 61.9, 47.5, 46.6, 42.0, 41.6, 38.7, 35.3, 34.8, 34.1. HRMS [M + Na]^+^ calcd for C_21_H_20_NaO_7_ 407.1101, found 407.1100.

### ((3*R*,4*R*)-3,4-bis(benzo[*d*][1,3]dioxol-5-ylmethyl)tetrahydrofuran-2-yl)methanol (**21**) and ((3a*R*,13a*R*)-6,7,10,11-bis(benzo[*d*][1,3]dioxol)-1,3,3a,4,13,13a-hexahydrodibenzo[4,5:6,7]cycloocta[1,2-c]furan-1-yl)methanol (**22**)

To a freshly distilled CHCl_3_ solution (10 mL) was added successively **20** (0.65 mmol, 1.0 equiv), a catalytic amount of InBr_3_ (0.03 mmol, 0.05 equiv), and Et_3_SiH (3.25 mmol, 5.0 eq). The solution was maintained at 60 °C for 2 h. During the stirring of the reaction mixture at 60 °C (bath temperature), the solution turned from colorless to yellow, then to white. The reaction was monitored by TLC until the consumption of the starting lactone. After the reaction, H_2_O (3 mL) was added, and the resulting orange suspension was stirred continuously until the disappearance of the color. The aqueous layer was partitioned with EtOAc (3 × 10 mL). The organic layer was separated, dried (Na_2_SO_4_), filtered, and the solvent was removed in vacuo. Flash chromatography (silica gel) of the crude reaction mixture afforded product **21** (143 mg, 60 %) and **22** (48 mg, 20 %) as a colorless oil. **21:**^1^H NMR (400 MHz, CDCl_3_) *δ* 6.68 (m, 2.4H), 6.54 (m, 4.8H), 5.91 (s, 4.8H), 3.90–4.10 (m, 0.2H), 3.82 (td, *J* = 8.0, 7.0, 2.0 Hz, 1H), 3.64–3.71 (m, 1.2H), 3.54–3.59 (m, 1.2H), 3.43 (dd, *J* = 11.8, 2.7 Hz, 1.2H), 3.23–3.37 (m, 1.2H), 2.41–2.65 (m, 4.8H), 2.13–2.25 (m, 1.2H), 1.86–1.92 (m, 1.2H); ^13^C NMR (100 MHz, CDCl_3_) *δ* 147.7, 147.6, 146.0, 145.8, 133.9, 133.4, 121.6, 121.4, 109.0, 108.9, 108.2, 108.1, 100.9, 85.6, 72.4, 64.0, 47.4, 47.1, 39.0. HRMS [M + Na]^+^ calcd for C_21_H_22_NaO_6_ 393.1314, found 393.1317. **22:**^1^H NMR (400 MHz, CDCl_3_) *δ* 6.96 (s, 1.2H), 6.85 (s, 1.2H), 6.62 (s, 1.2H), 6.48 (s, 1.2H), 5.70–6.03 (m, 4.8H), 4.15 (d, *J* = 7.9 Hz, 1.2H), 3.89 (dd, *J* = 10.4, 2.3 Hz, 1.2H), 3.53–3.78 (m, 2.4H), 3.19-3.41 (m, 1.2H), 2.55-2.98 (m, 4.8H), 2.45–2.50 (m, 1.2H), 2.08–2.16 (m, 1.2H); ^13^C NMR (100 MHz, CDCl_3_) *δ* 146.8, 146.3, 146.3, 145.7, 139.2, 135.3, 130.8, 127.5, 108.6, 108.0, 105.6, 105.4, 100.9, 100.7, 77.2, 73.9, 65.2, 47.8, 39.9, 38.3, 30.8, 28.2. HRMS [M + Na]^+^ calcd for C_21_H_20_NaO_6_ 391.1152, found 391.1143.

### (3*R*,4*R*)-3,4-bis(benzo[*d*][1,3]dioxol-5-ylmethyl)dihydrofuran-2(3*H*)-one (**1**)

To a solution of **21** (0.08 mmol, 1.0 eq) dry toluene (3 mL) was added PCC (0.40 mmol, 5.0 eq) and 4Å MS (30 mg), the mixture was stirred at 80 °C for 5 h. After the reaction, the mixture was filtered through a pad of Celite, and washed with EtOAc for 5 times and then concentrated. The resulting residue was purified by column chromatography (silica gel) to give (–)-hinokinin **1** (15 mg, 53 %) as a white solide. $\left[ \alpha \right]_{D}^{23}$ −31 (*c* 0.21, CHCl_3_), {lit. [5] $\left[ \alpha \right]_{D}^{21}$ −34 (*c* 2.85, CHCl_3_); lit. [21] $\left[ \alpha \right]_{D}^{26}$ −30 (*c* 0.99, CHCl_3_)}; ^1^H NMR (400 MHz, CDCl_3_) *δ* 6.44–6.73 (m, 6H), 5.92 (s, 4H), 4.11 (dd, J = 9.0, 6.7 Hz, 1H), 3.85 (dd, J = 9.2, 6.8 Hz, 1H), 2.97 (dd, J = 14.1, 5.0 Hz, 1H), 2.83 (dd, J = 14.1, 7.3 Hz, 1H), 2.48–2.65 (m, 2H), 2.45 (m, 2H). ^13^C NMR (100 MHz, CDCl_3_) *δ* 178.4, 147.8, 146.4, 146.3, 131.5, 131.2, 122.2, 121.5, 109.4, 108.8, 108.3, 108.2, 100.9, 71.1, 46.4, 41.2, 38.3, 34.8. The NMR data match those reported in the literature [3–5, 18, 21, 24, 26]. HRMS [M + Na]^+^ calcd for C_20_H_18_NaO_6_ 377.1001, found 377.1004; IR (KBr), 2958, 2924, 2855, 1761, 1503, 1489, 1443, 1257, 1189, 1098, 1036, 925, 864, 807, 771, 734, 676, 515.

## Electronic supplementary material

Supplementary material 1 (DOC 1509 kb)
