# Supplementary material for: Total Synthesis of Lignan Lactone (–)-Hinokinin
Source: Nat Prod Bioprospect. 2015 Oct 12;5(5):255–61. doi: 10.1007/s13659-015-0073-3 (PMC4607678; doi:10.1007/s13659-015-0073-3)
Supplement: Supplementary file 1 — Supplementary material 1 (DOC 1509 kb) [file 13659_2015_73_MOESM1_ESM.doc]

**Supporting Information**

**Total Synthesis of Lignan Lactone (−)-Hinokinin**

Qilong Zhou,^a^ Huijing Wang,^a^ Pei Tang,*^a^ Hao Song,^b^ Yong Qin*^b^

^a^ Innovative Drug Research Centre, Chongqing University, Chongqing 401331, China

^b^ Key Laboratory of Drug Targeting and Drug Delivery Systems of the Ministry of Education, West China School of Pharmacy, Sichuan University, Chengdu, 610041, China

E-mail: yongqin@scu.edu.cn; tangpei@cqu.edu.cn

**NMR spectrum of synthetic compounds**

^1^H-NMR spectrum of compound
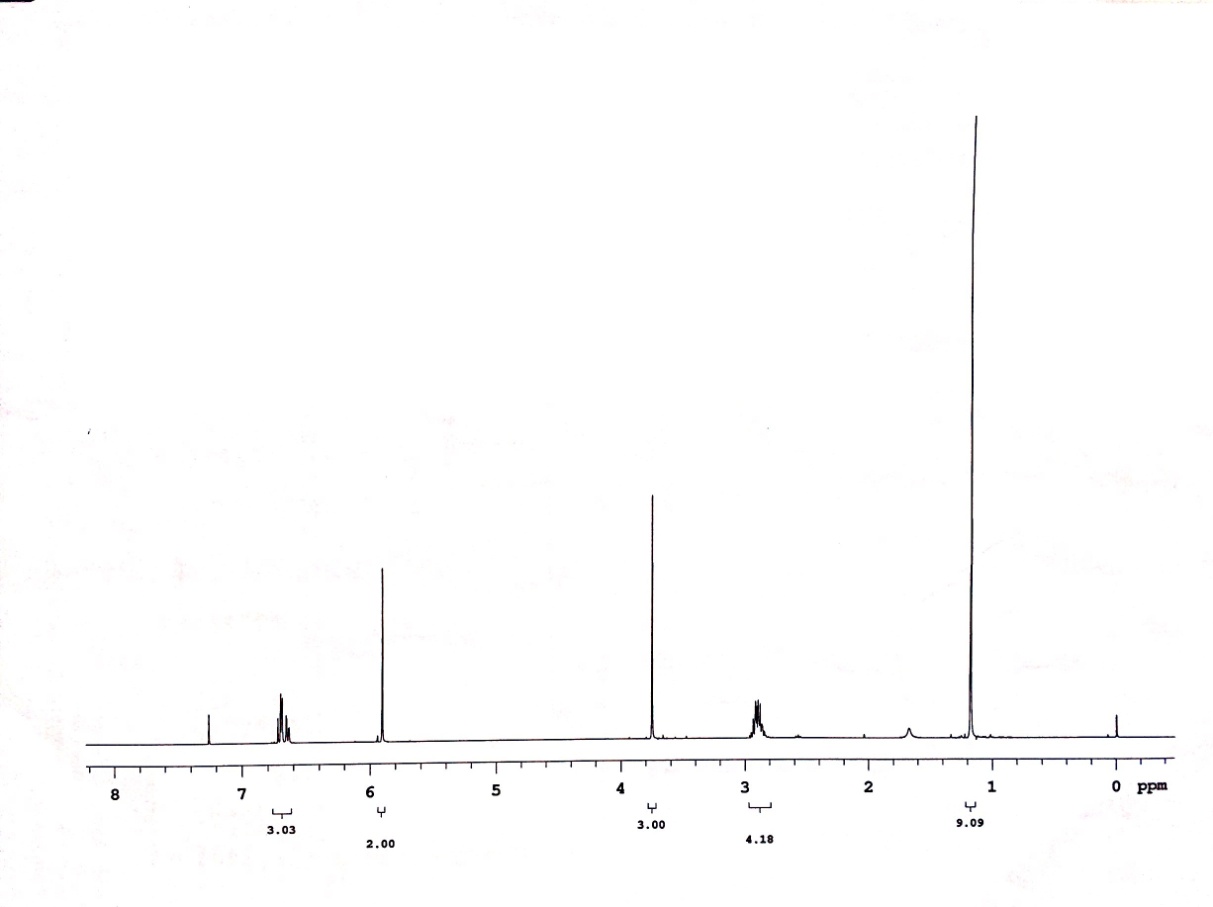
**14**


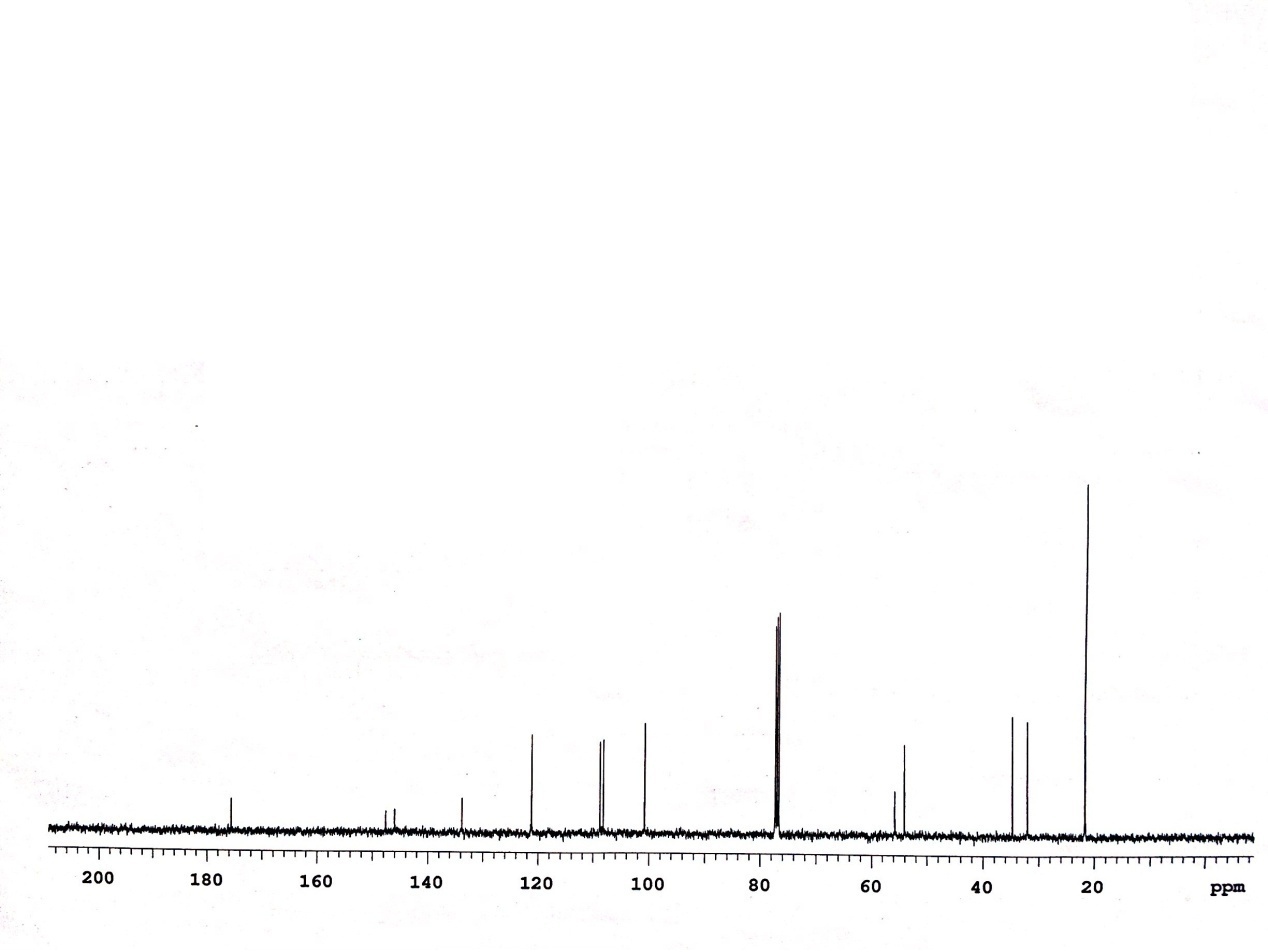
^13^C-NMR spectrum of compound **14**

^1^H-NMR spectrum of compound **1**
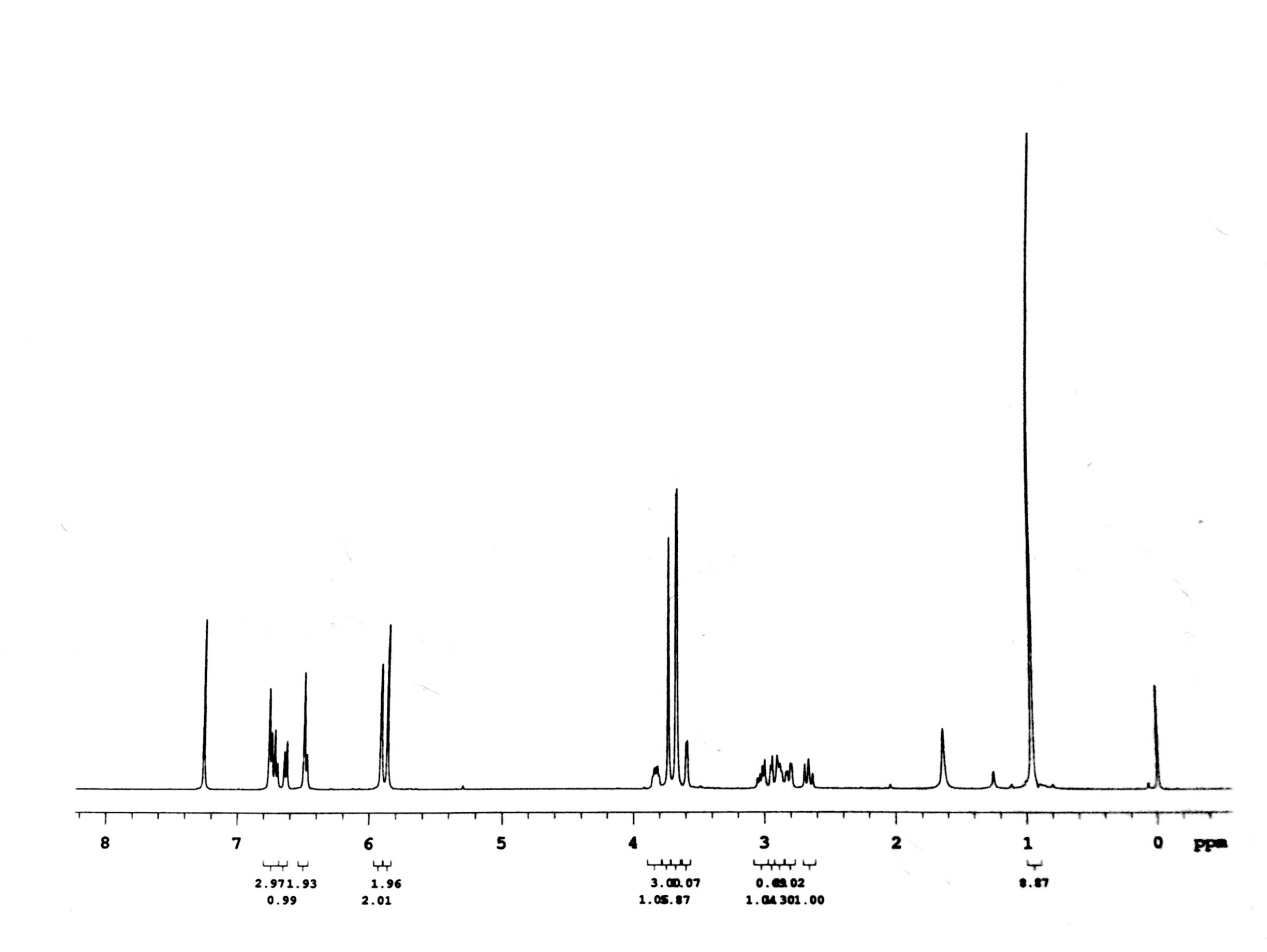
**7**

^13^C-NMR spectrum of compound **17**
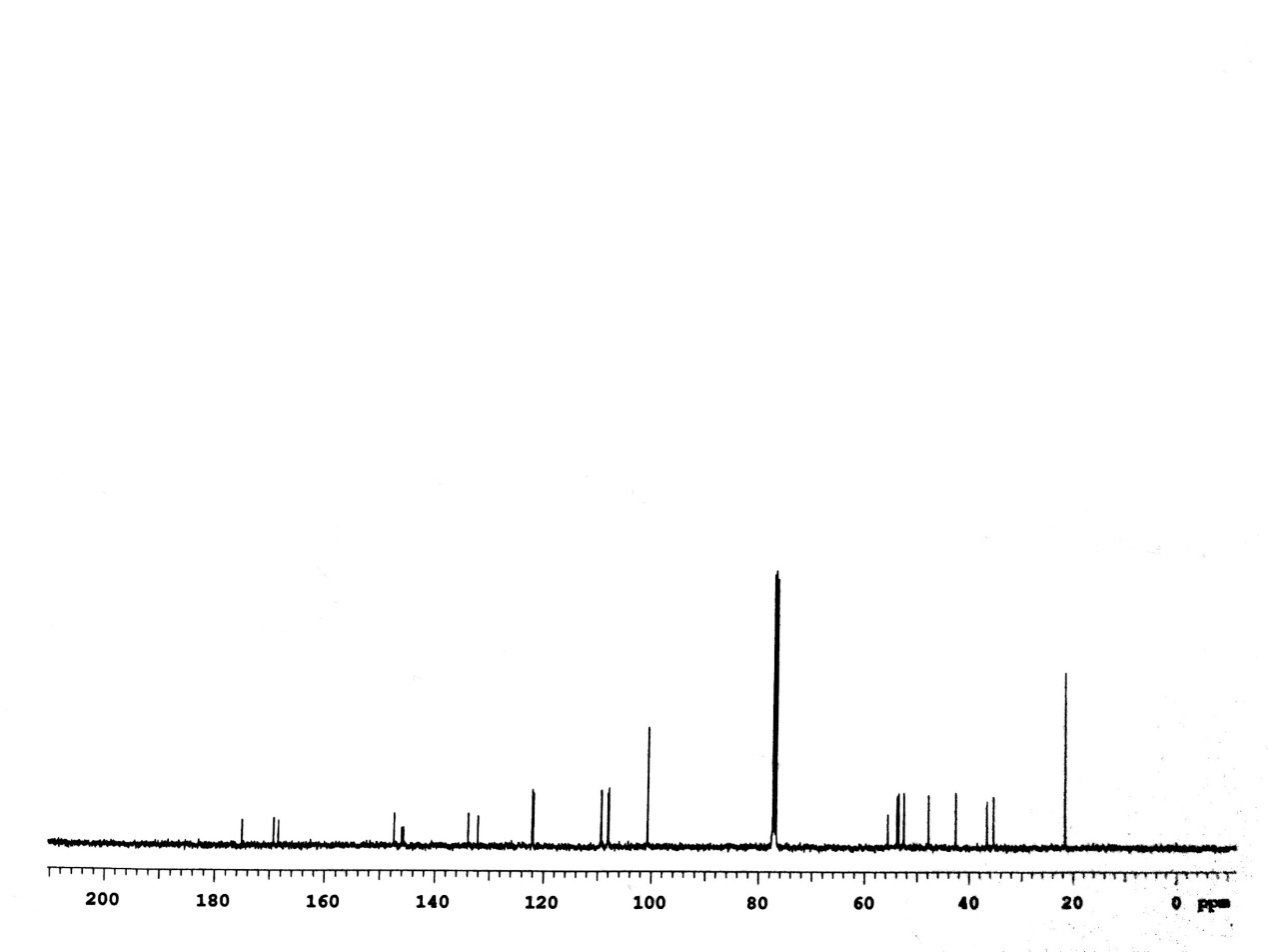


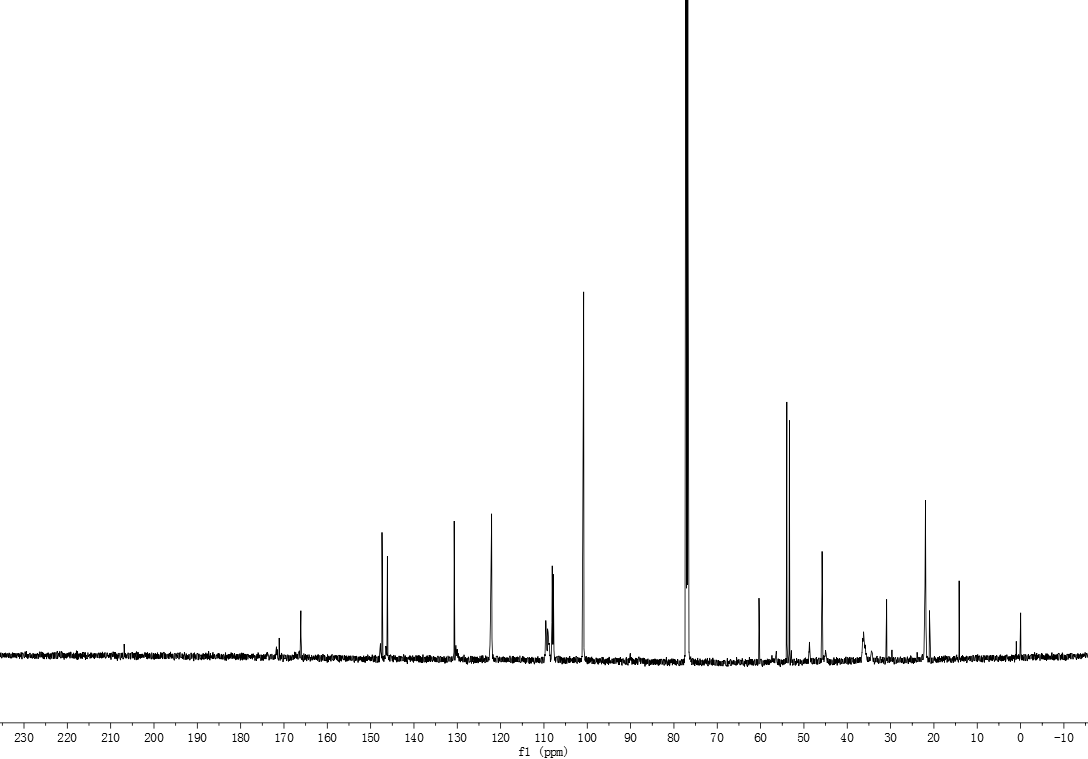
1H-NMR spectrum of compound
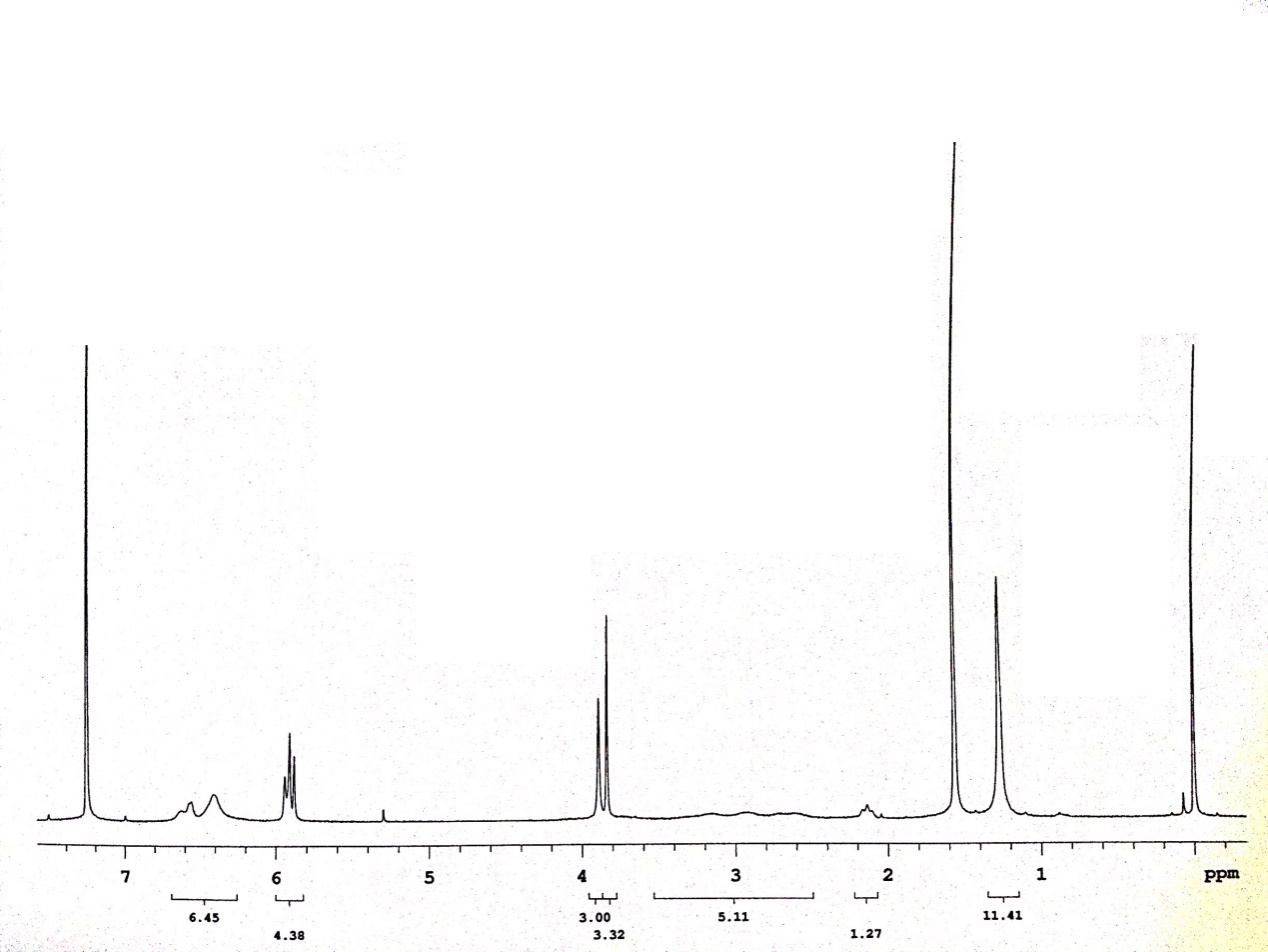
**16**

^13^C-NMR spectrum of compound **16**

^1^H-NMR spectrum of compound
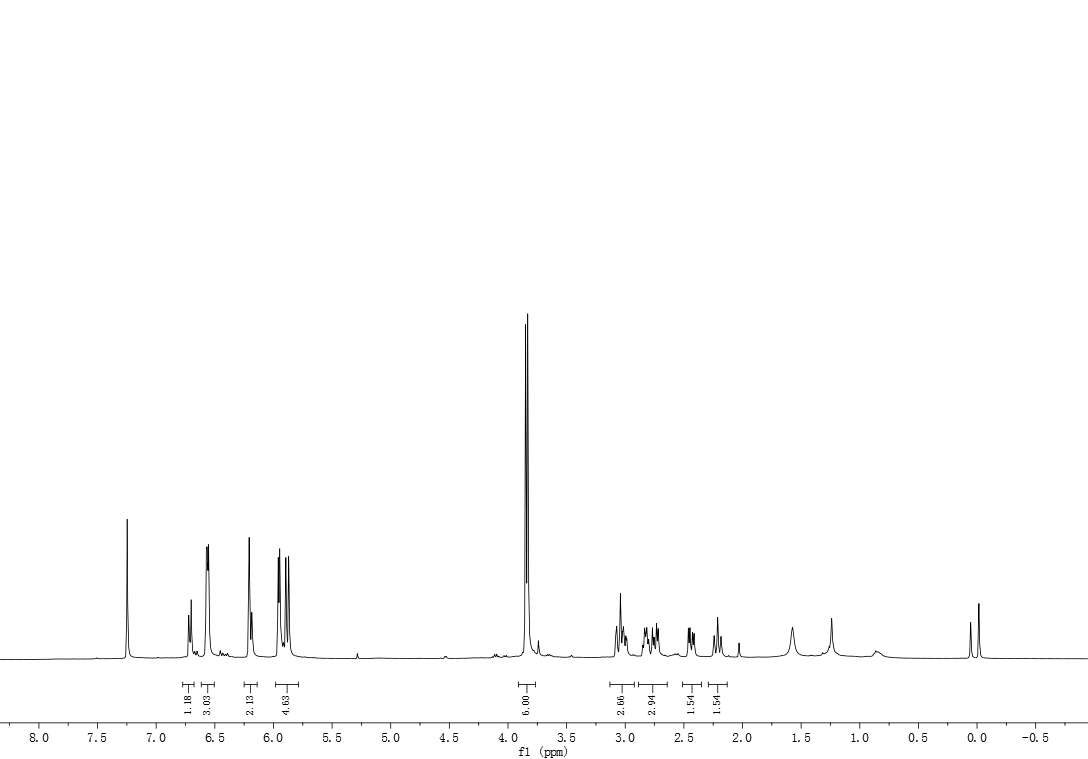

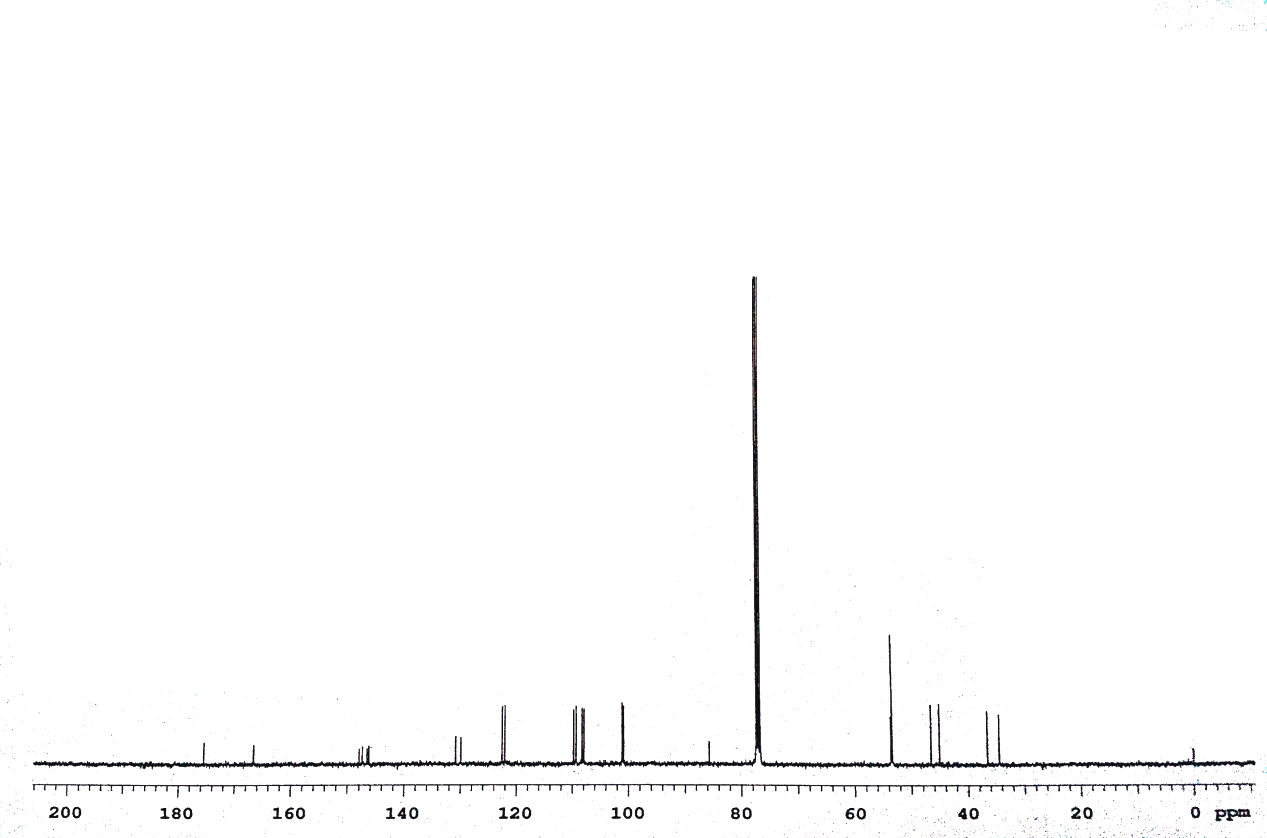
**18**

^13^C-NMR spectrum of compound **18**


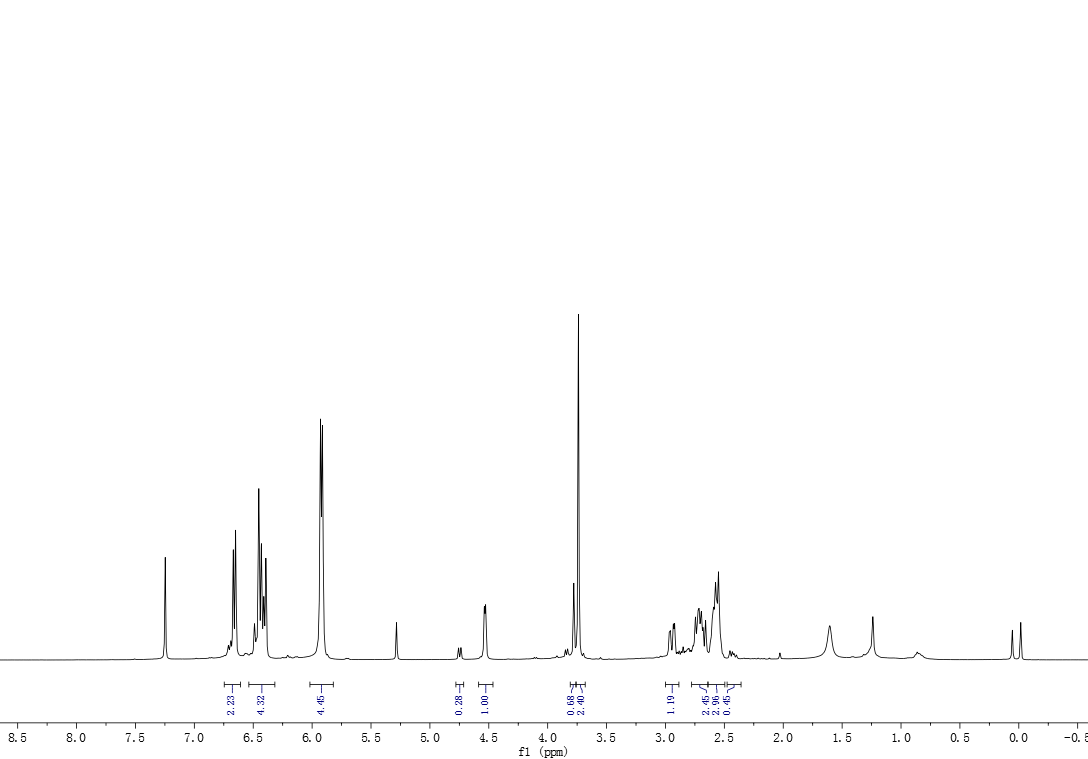

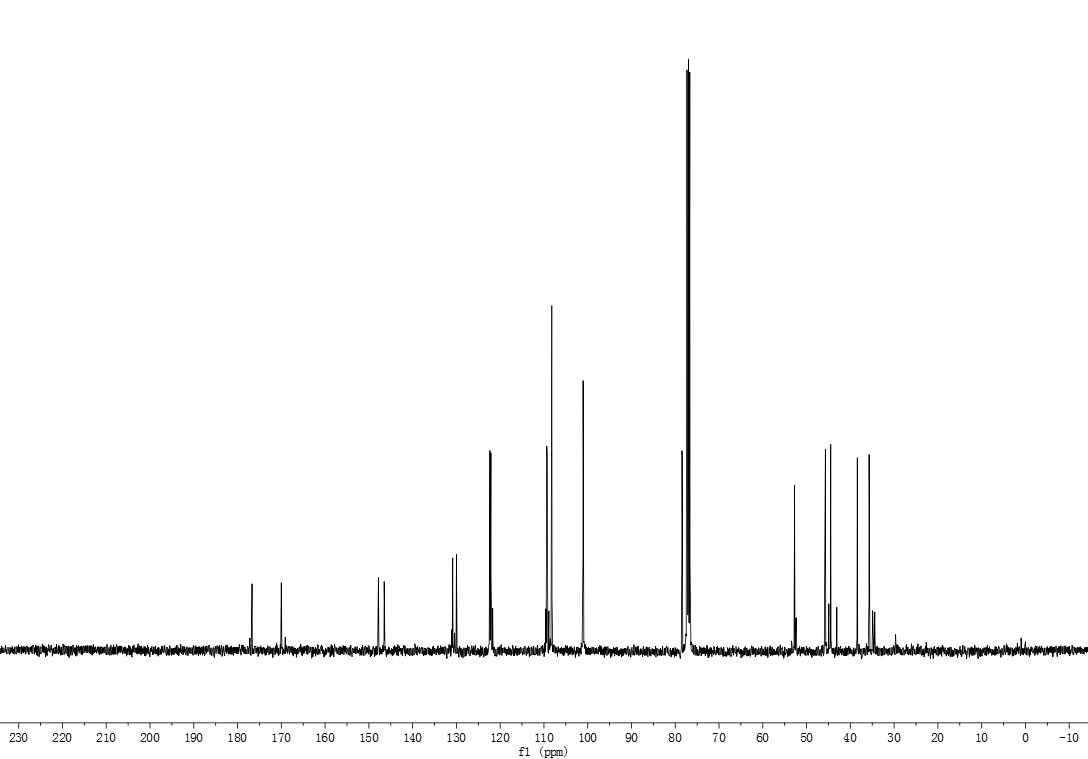
1H-NMR spectrum of compound **19**

^13^C-NMR spectrum of compound **19**

^1^H-NMR spectrum of compound
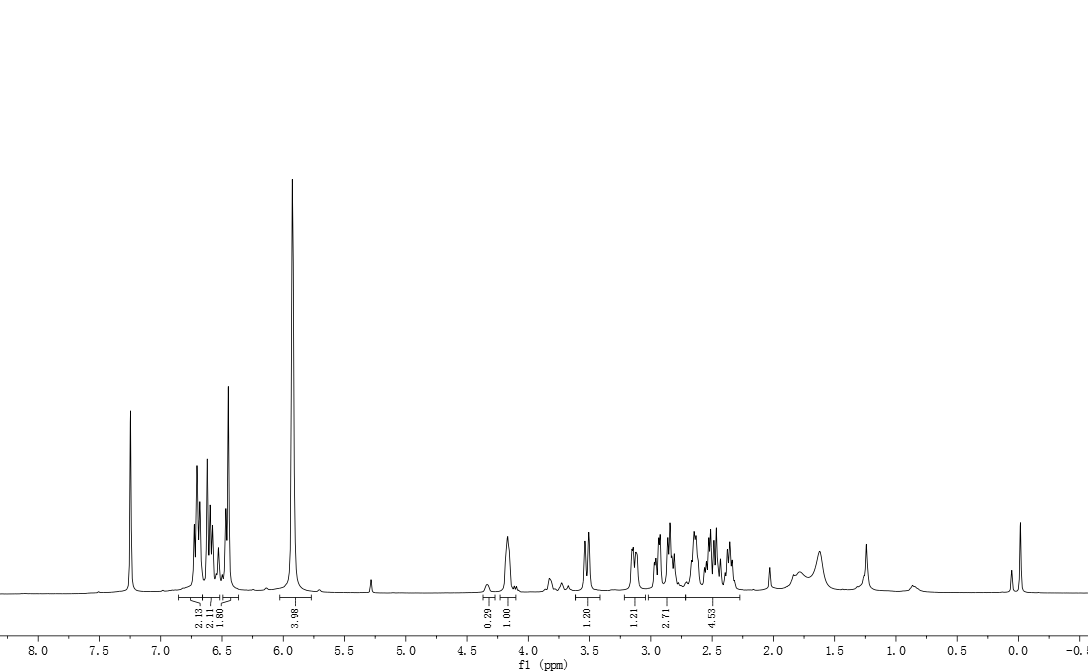
**20**

^13^C-NMR spectrum of compound
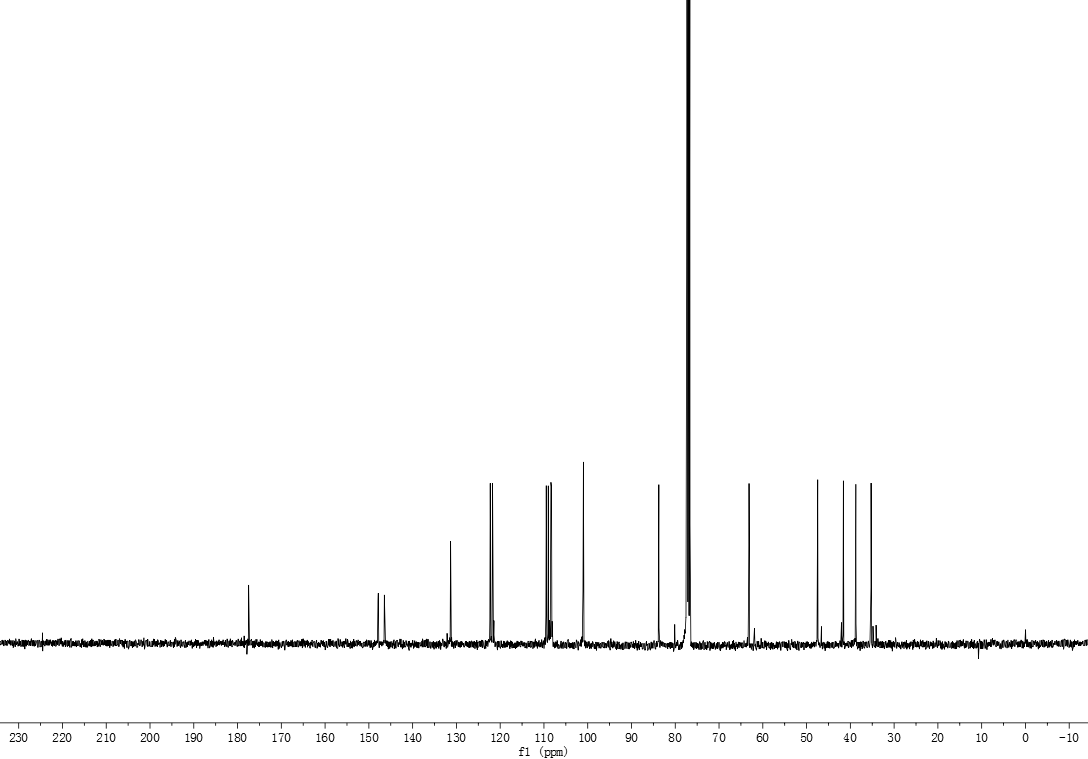
**20**

^1^H-NMR spectrum of compound
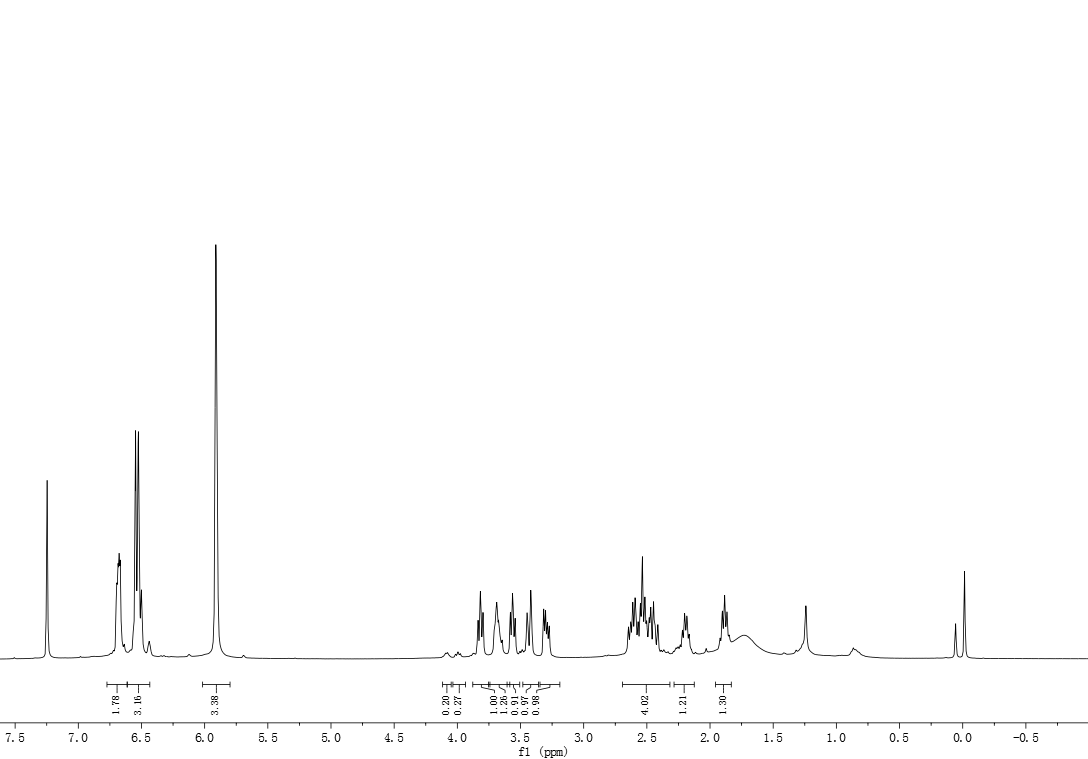

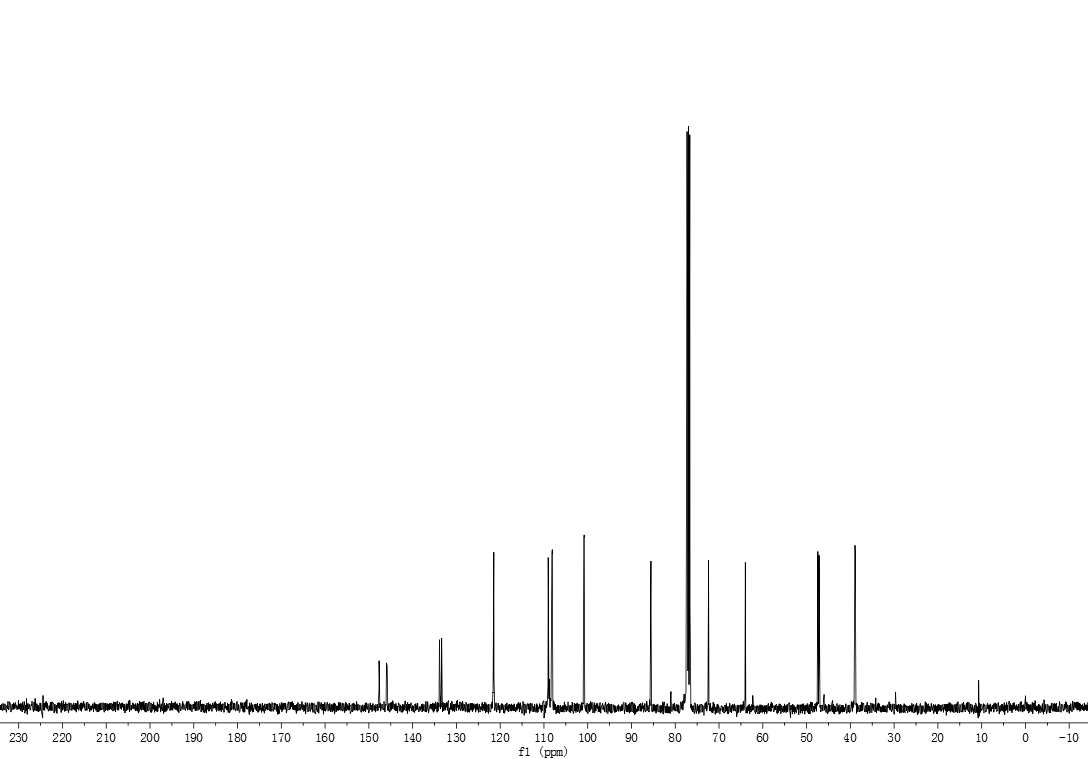
**21**

^13^C-NMR spectrum of compound **21**

^1^H-NMR spectrum of compound
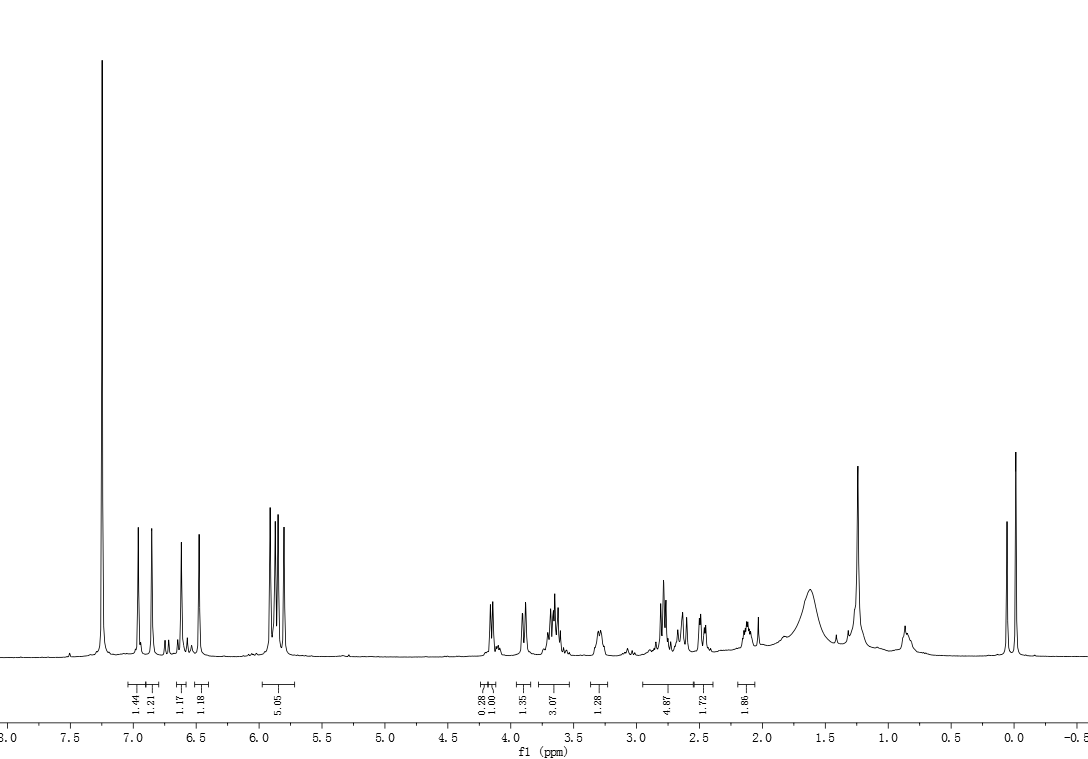
 **22**


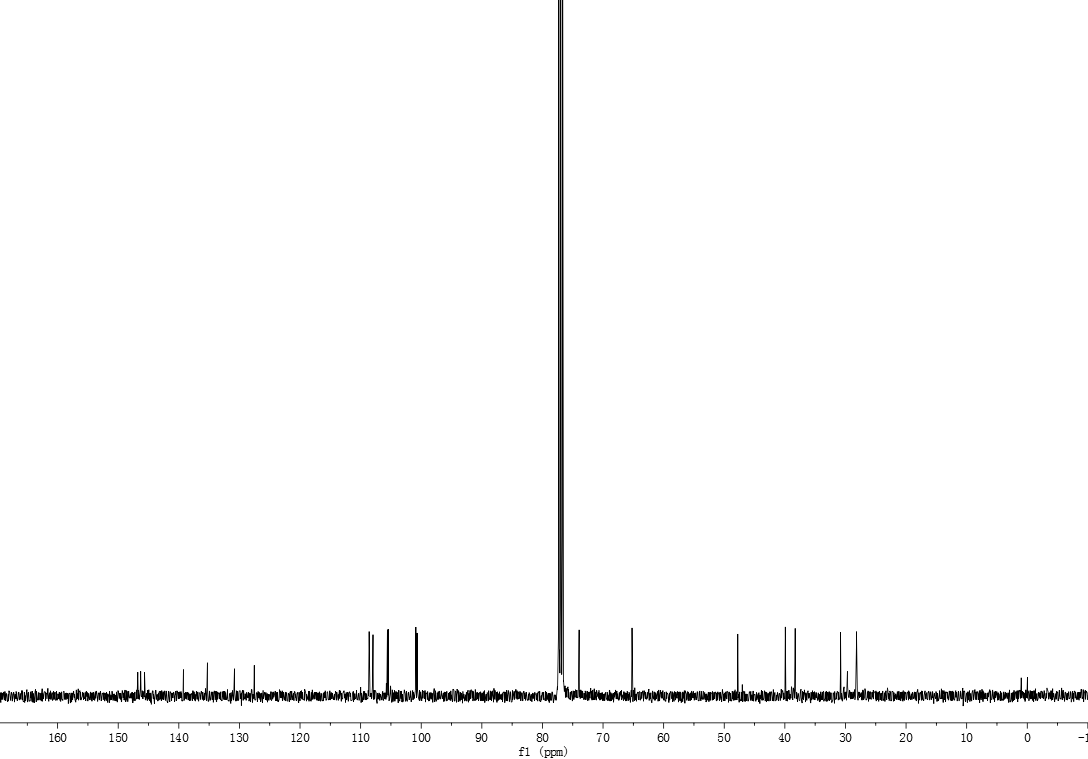


^13^C-NMR spectrum of compound **22**


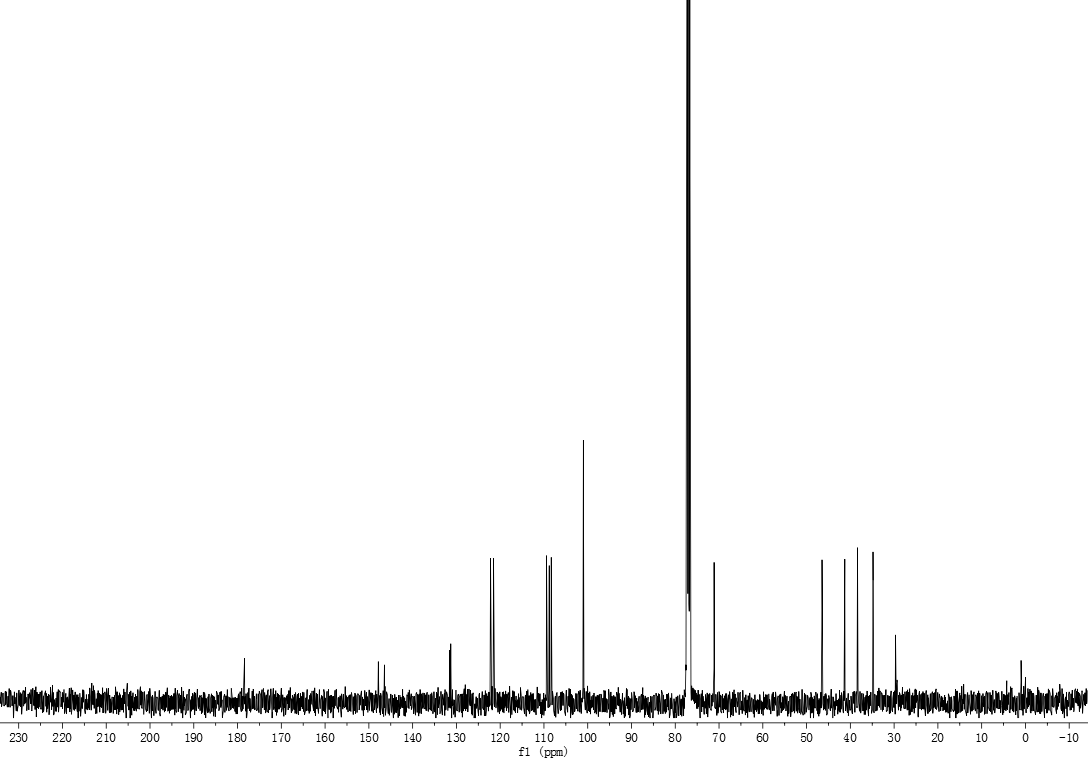
1H-NMR spectrum of
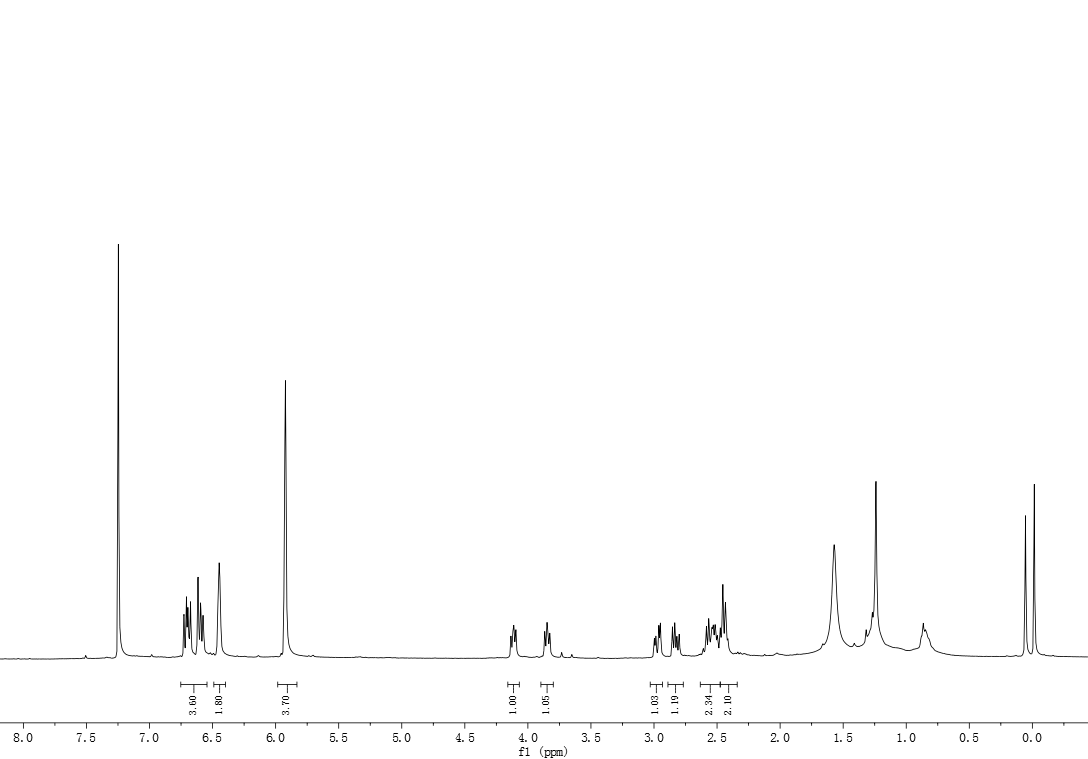
(−)-hinokinin (**1**)

^13^C-NMR spectrum of (−)-hinokinin (**1**)
